# Crystal structure of ethyl 2,4-di­chloro­quinoline-3-carboxyl­ate

**DOI:** 10.1107/S2056989015020587

**Published:** 2015-11-14

**Authors:** Alberto Cabrera, Luis D. Miranda, Héctor Reyes, Gerardo Aguirre, Daniel Chávez

**Affiliations:** a Centro de Graduados e Investigación en Química del Instituto, Tecnológico de, Tijuana, Apdo. Postal 1166, 22500, Tijuana, B.C., Mexico; b Instituto de Química, Universidad Nacional Autónoma de, México, Circuito, Exterior, S. N., Ciudad Universitaria, Coyoacán, México, D. F. 04510, México.

**Keywords:** crystal structure, quinnoline, human immunodeficiency virus (HIV), hydrogen bonding

## Abstract

In the crystal structure of the title compound, C_12_H_9_Cl_2_NO_2_, the mean planes through the quinoline and carboxyl­ate groups have r.m.s. deviations of 0.006 and 0.021 Å, respectively, and form a dihedral angle of 87.06 (19)°. In the crystal, mol­ecules are linked *via* very weak C—H⋯O hydrogen bonds, forming chains, which propagate along the *c-*axis direction.

## Related literature   

For the potential of related compounds in anti-HIV treatment, see: Maartens *et al.* (2014 ▸); Hopkins *et al.* (2004 ▸). For a related structure, see: Reyes *et al.* (2013 ▸)
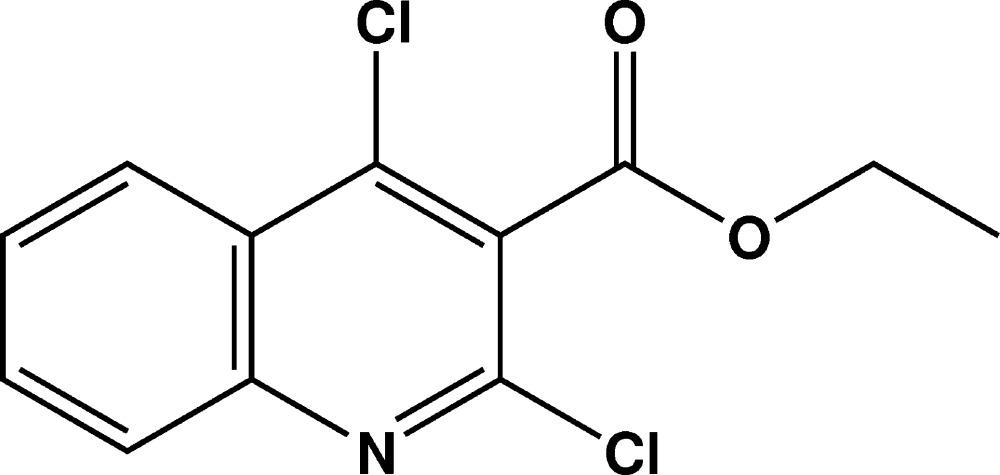


## Experimental   

### Crystal data  

C_12_H_9_Cl_2_NO_2_
*M* *_r_* = 270.10
Monoclinic, 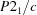

*a* = 8.5860 (4) Å
*b* = 19.9082 (11) Å
*c* = 7.1304 (4) Å
β = 100.262 (1)°
*V* = 1199.32 (11) Å^3^
*Z* = 4
Mo *K*α radiation
μ = 0.53 mm^−1^
*T* = 298 K
0.50 × 0.25 × 0.16 mm

### Data collection  

Bruker APEXII CCD area-detector diffractometer
6785 measured reflections
2197 independent reflections
1833 reflections with *I* > 2σ(*I*)
*R*_int_ = 0.024

### Refinement  

*R*[*F* ^2^ > 2σ(*F* ^2^)] = 0.035
*wR*(*F* ^2^) = 0.094
*S* = 1.05
2197 reflections
156 parameters
H-atom parameters constrained
Δρ_max_ = 0.22 e Å^−3^
Δρ_min_ = −0.21 e Å^−3^

### 

Data collection: *APEX2* (Bruker, 2012 ▸); cell refinement: *SAINT* (Bruker, 2012 ▸); data reduction: *SAINT*; program(s) used to solve structure: *SHELXTL* (Sheldrick, 2015 ▸); program(s) used to refine structure: *SHELXL2013* (Sheldrick, 2015 ▸); molecular graphics: *SHELXTL*; software used to prepare material for publication: *SHELXTL*.

## Supplementary Material

Crystal structure: contains datablock(s) I, global. DOI: 10.1107/S2056989015020587/nk2231sup1.cif

Structure factors: contains datablock(s) I. DOI: 10.1107/S2056989015020587/nk2231Isup2.hkl

Supporting information file. DOI: 10.1107/S2056989015020587/nk2231Isup3.cml

**Fig. 1.**
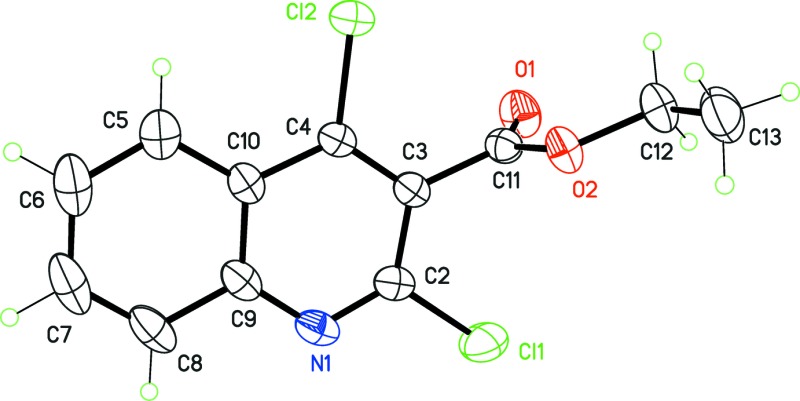
The asymmetric unit of the title compound with displacement ellipsoids drawn at the 50% probability level.

. DOI: 10.1107/S2056989015020587/nk2231fig1.tif
The asymmetric unit of the title compound with displacement ellipsoids drawn at the 50% probability level.

**Fig. 2.**
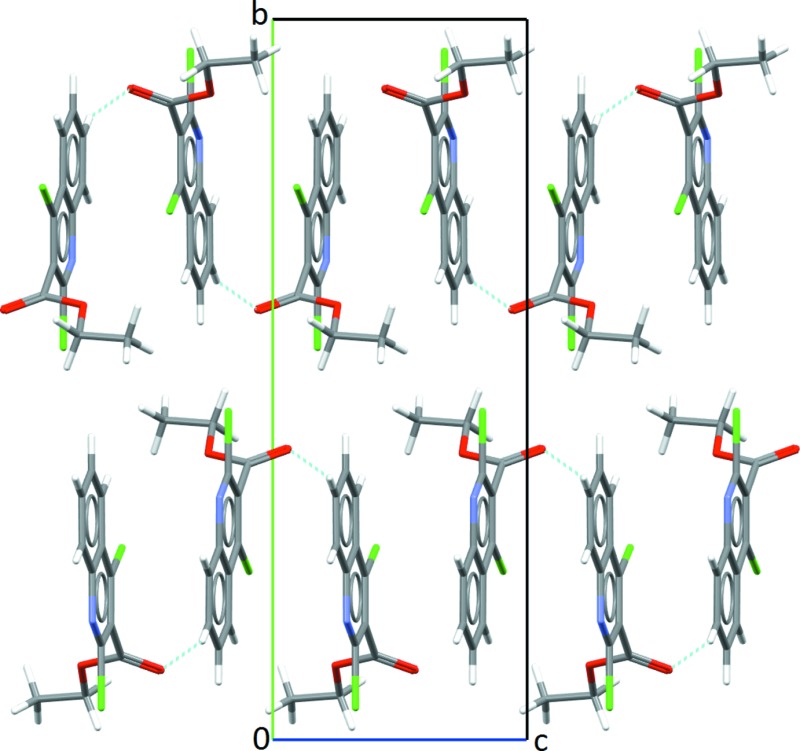
Crystal packing viewed along the a axis. The intermolecular C—H···O hydrogen bonds are shown as dashed lines.

. DOI: 10.1107/S2056989015020587/nk2231fig2.tif
Crystal packing viewed along the a axis. The inter­molecular C—H⋯O hydrogen bonds are shown as dashed lines.

CCDC reference: 1434378

Additional supporting information:  crystallographic information; 3D view; checkCIF report

## Figures and Tables

**Table 1:** Hydrogen-bond geometry (Å, °)

*D*—H⋯*A*	*D*—H	H⋯*A*	*D*⋯*A*	*D*—H⋯*A*
C7—H7⋯O1^i^	0.93	2.69	3.586 (3)	162

Symmetry code: (i) 
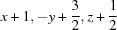
.

## supplementary crystallographic information

### S1. Chemical context

The HIV (human immunodeficiency virus) is responsible of the acquired immunodeficiency syndrome (AIDS). The actual treatment consists of a group of several drugs known as anti-retrovirals which inhibit important proteins for virus replication, including reverse transcriptase (Maartens *et al.*, 2014).

As part of the synthesis of promising compounds as anti-retrovirals, Stammers and coworkers, maintaining as base structure a quinolone core, found compounds that showed activity in the inhibition of the reverse transcriptase. They obtained a dibromide quinolone in C2/C4 as an inter­mediate. After that, they were able to remove the C2 bromine in an acetic acid solution and finally, the C4 bromine was substituted by alkyl-sulfurated compounds to get the final molecules which have been proven activity against HIV (Hopkins *et al.*, 2004),

As part of our ongoing research, we have synthesized different pyridin-2 (1*H*)-one analogues (Reyes *et al.*, 2013). In this work, we developed a methodology to obtain derivatives of quinolone, with saturated and unsaturated amines in in C4 with ethyl 4-hy­droxy-2-oxo-1,2-di­hydro­quinoline-3-carboxilate as a starting material. Chlorination of the quinolone core results in the dichlorinated compound ethyl 2,4-di­chloro­quinoline-3-carboxyl­ate with a yield of 70% and a crystal structure was obtained. The inter­mediate of inter­est, the ethyl 4-chloro-2-oxo-1,2-di­hydro­quinoline-3-carboxyl­ate, was obtained after treatment of the dichlorinated compound with an acetic acid solution following the methodology described by Stammers (Hopkins *et al.*, 2004) in >98% yield (see experimental section in supplementary material).

The structure of the compound is shown in Fig. 1. The two aromatic rings of quinoline are fused almost coaxially, with a dihedral angle between their planes of C8—C9—C10—C4= 179.21 (14)^o^. The carboxyl­ate group is in an anti­periplanar conformation to the quinoline with a torsion angle C12—O2—C11—C3 = -179.39 (15)°, and bonded to the quinoline over the plane C3—C11—O2 by 110.46 (14)^o^. The deviation of the bond length values for C2—Cl1= 1.745 (2) and C4— Cl 2= 1.7248 (16) from the standard values can be attributed for the sp2 hybridization of the quinoline ring. In the crystal, molecules are linked *via* O1—H7 inter­molecular hydrogen bonds forming chains propagating along the *c* axis direction. (Fig. 2).

### S2. Synthesis and crystallization

The synthesis of ethyl 2,4-di­chloro­quinoline-3-carboxyl­ate and ethyl 4-chloro-2-oxo-1,2-di­hydro­quinoline-3-carboxyl­ate includes reagents and reagent grade solvents, which were used without further purification. In a 100 mL round bottom flask equipped with a magnetic stirrer was placed 500 mg of ethyl 4-hy­droxy-2-oxo-1,2-di­hydro­quinoline-3-carboxyl­ate (2.15 mmol) and 1.96 g of benzyl­tri­ethyl­ammonium chloride in 15 mL of aceto­nitrile. Under continuous stirring, 0.88 mL of the phospho­ryl chloride (9.46 mmol) was added drop by drop. The mixture was stirred at 40^o^ C for 30 min and later at reflux for 1 h. Then, the solvent was evaporated and 15 mL of cold water was added and stirred for 1 h. Then a precipitate was obtained corresponding to a mixture of ethyl 2,4-di­chloro­quinoline-3-carboxyl­ate and ethyl 4-chloro-2-oxo-1,2-di­hydro­quinoline-3-carboxyl­ate. The precipitate was dissolved in 3 mL of di­chloro­methane-methanol (1:1, v/v). Partial evaporation leads to crystals (not suitable for X-ray diffraction) of ethyl 4-chloro-2-oxo-1,2-di­hydro­quinoline-3-carboxyl­ate (0.65 mmol, 30%). The rest corresponded to ethyl 2,4-di­chloro­quinoline-3-carboxyl­ate (1.5 mmol, 70%). The latter compound was placed in a round bottom flask of 50 mL containing acetic acid (10 mL) and water (5 mL). The mixture was stirred under reflux for 24 h. After cooling, the product was extracted with ethyl ether to afford ethyl 4-chloro-2-oxo-1,2-di­hydro­quinoline-3-carboxyl­ate (>98%).

Ethyl 2,4-di­chloro­quinoline-3-carboxyl­ate: m.p. 83-85 °C. ^1^H RMN (DMSO-*d6*): δ 8.27 (dd, *J* = 8.3, 0.6 Hz, H-5), 8.09 (dd, *J* = 8.3, 0.6 Hz, H-8), 8.03 (ddd, *J* = 7.7, 6.9, 1.4 Hz, H-7), 7.89 (ddd, *J* = 7.7, 6.9, 1.4 Hz, H-6) 4.51 (q, *J* = 7.1 Hz, COOCH_2_CH_3_), 1.39 (t, *J* = 7.1 Hz, COOCH_2_CH_3_). ^13^C RMN (DMSO-*d*6): δ 163.5, 147.2, 144.7, 141.1, 133.7, 130.2, 129.1, 127.1, 124.9, 124.3, 63.4, 14.3. IEMS *m/e* (int. *rel*): [M]^+^ 269 (32), [M]^+^+2 271 (21), [M]^+^+4 273 (3), 241 (26), 223 (100), 195 (17), 161 (28) amu.

Crystals of the title compound suitable for X-ray diffraction were obtained by dissolving 15 mg of ethyl 2,4-di­chloro­quinoline-3-carboxyl­ate in 0.5 mL of ethanol-di-ethyl ether (1:1, v / v) and placing the solution in a glass vial. The solution was allowed to stand at room temperature for 7 days and the crystals formed were filtered.

### S3. Refinement details

The C-bound H atoms were positioned geometrically and refined using a riding model with d(C—H) = 1.00 Å, U_iso_ = 1.2Ueq(C) for Csp^3^—H, d(C—H) = 0.99 Å, U_iso_ = 1.2Ueq(C) for CH_2_ groups, d(C—H) = 0.95 Å, U_iso_ = 1.2Ueq(C) for aromatic C—H.

### Crystal data

**Table:**

C_12_H_9_Cl_2_NO_2_	*F*(000) = 552
*M**_r_* = 270.10	*D*_x_ = 1.496 Mg m^−^^3^
Monoclinic, *P*2_1_/*c*	Mo *K*α radiation, λ = 0.71073 Å
*a* = 8.5860 (4) Å	Cell parameters from 4843 reflections
*b* = 19.9082 (11) Å	θ = 2.4–25.4°
*c* = 7.1304 (4) Å	µ = 0.53 mm^−^^1^
β = 100.262 (1)°	*T* = 298 K
*V* = 1199.32 (11) Å^3^	Prism, colourless
*Z* = 4	0.50 × 0.25 × 0.16 mm

### Data collection

**Table:**

Bruker APEXII CCD area-detector diffractometer	*R*_int_ = 0.024
Detector resolution: 0.83 pixels mm^-1^	θ_max_ = 25.4°, θ_min_ = 2.1°
ω scans	*h* = −10→10
6785 measured reflections	*k* = −23→23
2197 independent reflections	*l* = −8→8
1833 reflections with *I* > 2σ(*I*)	

### Refinement

**Table:**

Refinement on *F*^2^	Hydrogen site location: inferred from neighbouring sites
Least-squares matrix: full	H-atom parameters constrained
*R*[*F*^2^ > 2σ(*F*^2^)] = 0.035	*w* = 1/[σ^2^(*F*_o_^2^) + (0.052*P*)^2^ + 0.1257*P*] where *P* = (*F*_o_^2^ + 2*F*_c_^2^)/3
*wR*(*F*^2^) = 0.094	(Δ/σ)_max_ = 0.001
*S* = 1.05	Δρ_max_ = 0.22 e Å^−^^3^
2197 reflections	Δρ_min_ = −0.21 e Å^−^^3^
156 parameters	Extinction correction: *SHELXL2013* (Sheldrick, 2015), Fc^*^=kFc[1+0.001xFc^2^λ^3^/sin(2θ)]^-1/4^
0 restraints	Extinction coefficient: 0.010 (2)

### Special details

**Table:**

Geometry. All e.s.d.'s (except the e.s.d. in the dihedral angle between two l.s. planes) are estimated using the full covariance matrix. The cell e.s.d.'s are taken into account individually in the estimation of e.s.d.'s in distances, angles and torsion angles; correlations between e.s.d.'s in cell parameters are only used when they are defined by crystal symmetry. An approximate (isotropic) treatment of cell e.s.d.'s is used for estimating e.s.d.'s involving l.s. planes.

### Fractional atomic coordinates and isotropic or equivalent isotropic displacement parameters (Å^2^)

**Table:**

	*x*	*y*	*z*	*U*_iso_*/*U*_eq_	
Cl1	0.85142 (7)	0.95554 (3)	0.67524 (10)	0.0847 (2)	
Cl2	0.49224 (5)	0.73357 (2)	0.59388 (7)	0.05637 (19)	
O1	0.48324 (17)	0.90370 (7)	0.44065 (18)	0.0701 (4)	
O2	0.48524 (14)	0.89699 (6)	0.75480 (17)	0.0565 (3)	
N1	0.96891 (17)	0.83536 (9)	0.7101 (2)	0.0567 (4)	
C2	0.8376 (2)	0.86809 (9)	0.6757 (2)	0.0514 (4)	
C3	0.68405 (18)	0.84036 (8)	0.6379 (2)	0.0435 (4)	
C4	0.67539 (18)	0.77181 (8)	0.6378 (2)	0.0415 (4)	
C5	0.8137 (3)	0.66071 (10)	0.6754 (3)	0.0621 (5)	
H5	0.7187	0.6372	0.6505	0.075*	
C6	0.9535 (3)	0.62696 (13)	0.7135 (3)	0.0816 (7)	
H6	0.9531	0.5802	0.7143	0.098*	
C7	1.0976 (3)	0.66121 (14)	0.7514 (3)	0.0838 (8)	
H7	1.1918	0.6371	0.7779	0.101*	
C8	1.1014 (2)	0.72928 (13)	0.7498 (3)	0.0705 (6)	
H8	1.1981	0.7516	0.7748	0.085*	
C9	0.9593 (2)	0.76660 (10)	0.7105 (2)	0.0511 (5)	
C10	0.81366 (19)	0.73162 (9)	0.6738 (2)	0.0451 (4)	
C11	0.5396 (2)	0.88399 (9)	0.5963 (2)	0.0478 (4)	
C12	0.3434 (2)	0.93853 (11)	0.7367 (3)	0.0674 (5)	
H12A	0.2575	0.9182	0.6481	0.081*	
H12B	0.3638	0.9828	0.6897	0.081*	
C13	0.3010 (3)	0.94358 (13)	0.9279 (3)	0.0858 (7)	
H13A	0.2721	0.9000	0.9681	0.129*	
H13B	0.2133	0.9738	0.9235	0.129*	
H13C	0.3900	0.9602	1.0166	0.129*	

### Atomic displacement parameters (Å^2^)

**Table:**

	*U*^11^	*U*^22^	*U*^33^	*U*^12^	*U*^13^	*U*^23^
Cl1	0.0773 (4)	0.0613 (3)	0.1107 (5)	−0.0197 (3)	0.0034 (3)	−0.0015 (3)
Cl2	0.0445 (3)	0.0641 (3)	0.0597 (3)	−0.01024 (19)	0.0070 (2)	−0.0018 (2)
O1	0.0713 (9)	0.0848 (10)	0.0506 (8)	0.0213 (7)	0.0009 (7)	0.0096 (7)
O2	0.0498 (7)	0.0681 (8)	0.0529 (7)	0.0183 (6)	0.0129 (6)	0.0081 (6)
N1	0.0395 (8)	0.0795 (11)	0.0508 (9)	−0.0050 (7)	0.0070 (6)	−0.0022 (8)
C2	0.0455 (10)	0.0606 (11)	0.0475 (10)	−0.0066 (8)	0.0064 (8)	−0.0016 (8)
C3	0.0393 (9)	0.0544 (10)	0.0367 (8)	0.0011 (7)	0.0069 (7)	0.0000 (7)
C4	0.0377 (8)	0.0548 (10)	0.0325 (8)	−0.0016 (7)	0.0073 (6)	−0.0002 (7)
C5	0.0719 (13)	0.0630 (12)	0.0520 (11)	0.0133 (10)	0.0128 (9)	0.0027 (9)
C6	0.0980 (19)	0.0785 (15)	0.0680 (14)	0.0429 (14)	0.0143 (13)	0.0066 (11)
C7	0.0768 (16)	0.118 (2)	0.0564 (13)	0.0554 (16)	0.0110 (11)	0.0060 (13)
C8	0.0445 (11)	0.120 (2)	0.0476 (11)	0.0239 (11)	0.0085 (8)	−0.0009 (11)
C9	0.0412 (9)	0.0793 (13)	0.0337 (9)	0.0101 (8)	0.0090 (7)	−0.0001 (8)
C10	0.0451 (9)	0.0608 (11)	0.0301 (8)	0.0100 (8)	0.0088 (7)	0.0016 (7)
C11	0.0440 (9)	0.0499 (9)	0.0481 (10)	−0.0006 (7)	0.0042 (8)	0.0018 (8)
C12	0.0544 (11)	0.0706 (12)	0.0778 (13)	0.0229 (10)	0.0136 (10)	0.0079 (11)
C13	0.0641 (14)	0.1089 (19)	0.0878 (16)	0.0215 (13)	0.0227 (12)	−0.0130 (14)

### Geometric parameters (Å, º)

**Table:**

Cl1—C2	1.745 (2)	C6—C7	1.396 (4)
Cl2—C4	1.7248 (16)	C6—H6	0.9300
O1—C11	1.195 (2)	C7—C8	1.356 (4)
O2—C11	1.323 (2)	C7—H7	0.9300
O2—C12	1.459 (2)	C8—C9	1.413 (3)
N1—C2	1.287 (2)	C8—H8	0.9300
N1—C9	1.371 (3)	C9—C10	1.414 (2)
C2—C3	1.411 (2)	C12—C13	1.476 (3)
C3—C4	1.367 (2)	C12—H12A	0.9700
C3—C11	1.500 (2)	C12—H12B	0.9700
C4—C10	1.417 (2)	C13—H13A	0.9600
C5—C6	1.360 (3)	C13—H13B	0.9600
C5—C10	1.412 (3)	C13—H13C	0.9600
C5—H5	0.9300		
			
C11—O2—C12	116.84 (14)	C9—C8—H8	119.8
C2—N1—C9	117.06 (15)	N1—C9—C8	118.38 (18)
N1—C2—C3	126.55 (18)	N1—C9—C10	122.85 (15)
N1—C2—Cl1	116.62 (14)	C8—C9—C10	118.77 (19)
C3—C2—Cl1	116.83 (14)	C5—C10—C9	119.45 (16)
C4—C3—C2	116.09 (15)	C5—C10—C4	124.43 (17)
C4—C3—C11	122.35 (14)	C9—C10—C4	116.12 (16)
C2—C3—C11	121.55 (15)	O1—C11—O2	125.64 (16)
C3—C4—C10	121.33 (15)	O1—C11—C3	123.90 (16)
C3—C4—Cl2	119.25 (12)	O2—C11—C3	110.46 (14)
C10—C4—Cl2	119.42 (13)	O2—C12—C13	107.24 (16)
C6—C5—C10	119.7 (2)	O2—C12—H12A	110.3
C6—C5—H5	120.2	C13—C12—H12A	110.3
C10—C5—H5	120.2	O2—C12—H12B	110.3
C5—C6—C7	121.2 (2)	C13—C12—H12B	110.3
C5—C6—H6	119.4	H12A—C12—H12B	108.5
C7—C6—H6	119.4	C12—C13—H13A	109.5
C8—C7—C6	120.5 (2)	C12—C13—H13B	109.5
C8—C7—H7	119.7	H13A—C13—H13B	109.5
C6—C7—H7	119.7	C12—C13—H13C	109.5
C7—C8—C9	120.4 (2)	H13A—C13—H13C	109.5
C7—C8—H8	119.8	H13B—C13—H13C	109.5

### Hydrogen-bond geometry (Å, º)

**Table:**

*D*—H···*A*	*D*—H	H···*A*	*D*···*A*	*D*—H···*A*
C7—H7···O1^i^	0.93	2.69	3.586 (3)	162

Symmetry code: (i) *x*+1, −*y*+3/2, *z*+1/2.

## References

[bb1] Bruker (2012). *APEX2* and *SAINT*. Bruker AXS Inc., Madison, Wisconsin, USA.

[bb2] Hopkins, A. L., Ren, J., Milton, J., Hazen, R. J., Chan, J. H., Stuart, D. I. & Stammers, D. K. (2004). *J. Med. Chem.* **47**, 5912–5922. 10.1021/jm040071z 15537346

[bb3] Maartens, G., Celum, C. & Lewin, S. R. (2014). *Lancet*, **384**, 258–271. 10.1016/S0140-6736(14)60164-1 24907868

[bb4] Reyes, H., Aguirre, G. & Chávez, D. (2013). *Acta Cryst.* E**69**, o1534. 10.1107/S1600536813024240 PMC3790402 24098221

[bb5] Sheldrick, G. M. (2015). *Acta Cryst.* C**71**, 3–8.

