# Supplementary figures and images for: Crystal structure of ethyl 2,4-di­chloro­quinoline-3-carboxyl­ate
Source: Acta Crystallogr E Crystallogr Commun. 2015 Nov 14;71(Pt 12):o939. doi: 10.1107/S2056989015020587 (PMC4719891; doi:10.1107/S2056989015020587)

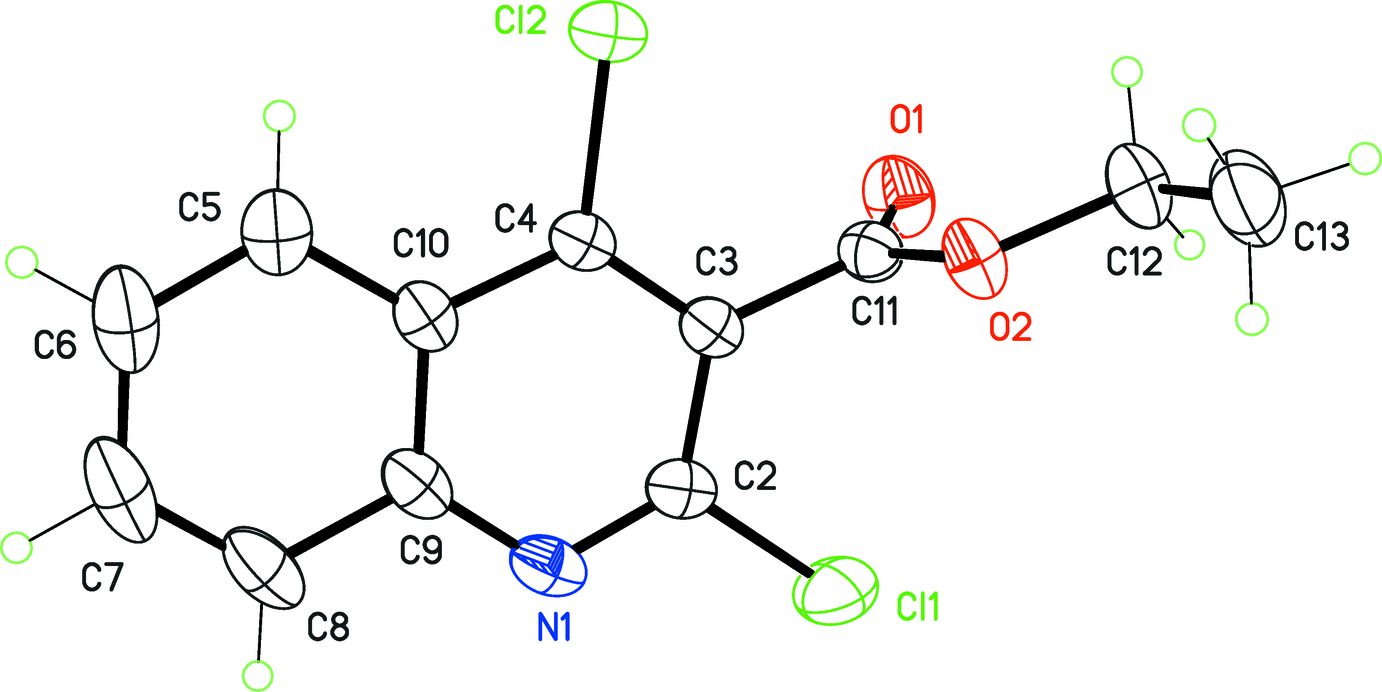

Supplement: Supplementary file 4 [file e-71-0o939-fig1.tif]

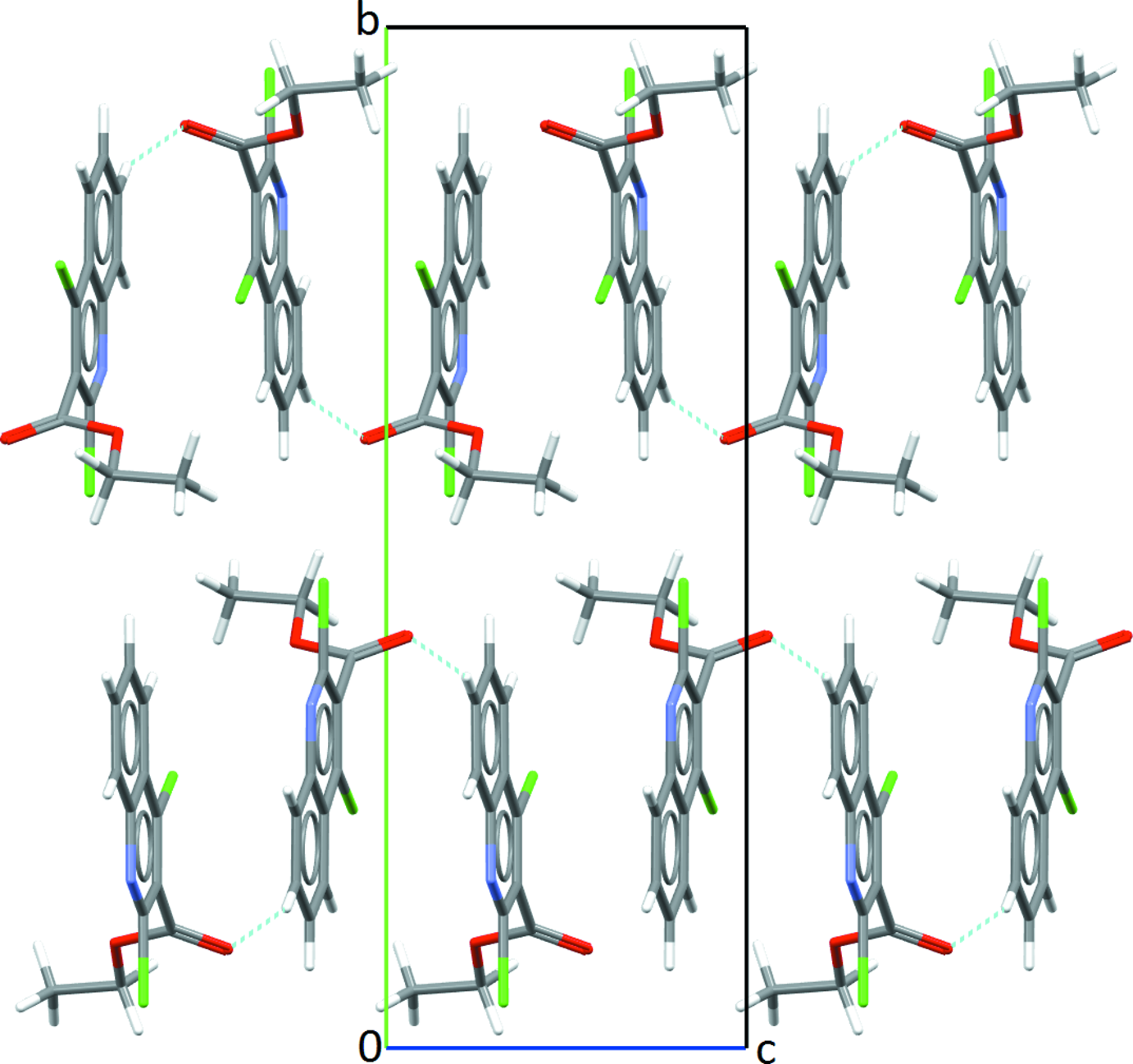

Supplement: Supplementary file 5 [file e-71-0o939-fig2.tif]
